# Treatment of Liver Cancer: Role of the Traditional Mongolian Medicine

**DOI:** 10.1155/2022/6535977

**Published:** 2022-02-14

**Authors:** Xiaomei Bao, Lu Chen, Yiman Liu, Hua Sheng, Kailong Wang, Yanming Luo, Tongling Qin, Ying Liu, Yuling Qiu

**Affiliations:** ^1^State Key Laboratory of Component-based Chinese Medicine, Tianjin University of Traditional Chinese Medicine, Tianjin, China; ^2^School of Pharmacy, Inner Mongolia Medical University, Hohhot, China; ^3^School of Pharmacy, Tianjin Medical University, Tianjin, China

## Abstract

Liver cancer is an extraordinarily heterogeneous malignancy with relatively high mortality and increasing incidence rate among the so far identified cancers. Improvements in liver cancer therapy have been made in the past decades, but therapeutics against liver cancer are still limited. Traditional Mongolian Medicine, formed and developed by the Mongolian people to maintain health in the medical practice of fighting against diseases, has been recognized as one of the key components of the world healthcare system. Traditional Mongolian Medicine has been used to treat various malignancies, including liver cancer, for a long time in Asia and its advantages have become more and more apparent. Herein, this review made a comprehensive summary of Traditional Mongolian Medicine, including the ideas in the liver cancer treatment, sources of medicines or prescriptions, traditional applications, modern pharmacological research, chemical structure and mechanisms of several monomer compounds isolated from Traditional Mongolian Medicine, with a view to finding promising drugs against liver cancer and expanding the clinical application of Traditional Mongolian Medicine in liver cancer therapy.

## 1. Introduction

Liver cancer has an insidious onset. Even though many patients have been effectively diagnosed and treated in the early stage of the disease, the recurrence rate is still high [1, 2]. Especially for patients with advanced disease, the prognosis is not optimistic. If cancer spreads to the peripheral lymph nodes, the patient's 5-year survival rate is only 11%. The current treatment methods for liver cancer include surgery, radiotherapy, minimally invasive treatment (radiofrequency ablation, argon helium knife, microwave ablation, interventional therapy), biological immunotherapy, traditional Chinese medicine, and diet therapy. Surgery is the preferred treatment for liver cancer. However, only 20%–30% of patients can get surgical resection because most patients have basic liver disease, or most of them have reached the advanced stage at the time of diagnosis [3, 4]. Although the clinical treatment of liver cancer has been improved in the past years, the overall survival remains unsatisfactory. Especially, patients in the advanced stage still have limited treatment options. Therefore, it is of great significance to further explore the occurrence and development mechanism of liver cancer and find new therapeutics.

Traditional Chinese ethnic medicine is the creation and accumulation of every ethnic minority through their long histories [5]. As an indispensable part of traditional Chinese medicine, Mongolian medicine is an important traditional medicine formed and developed by the Mongolian people in the medical practice of fighting against diseases for a long time and has its own unique diagnostic methods [6]. Mongolian medicine absorbs and digests the theory of Tibetan medicine and other ethnic medicine. It combines its own theories and experience to create the Mongolian medicine system with characteristics of Mongolian culture and history. Its development has received the attention of the Chinese government and the support of relevant policies [7].

As the only remaining historical documents, Mongolian medical literature has a complete medical theory system and is still used in clinical practice. The classic books of Mongolian medicine include Ren yao bai jing jian, the Four Medical Tantras, and Classic Canon of Mongolian Materia Medica. Ren yao bai jing jian is the foundation of Mongolian medicine, containing 390 kinds of Mongolian medicines [8]. The Four Medical Tantras is a comprehensive work on Tibetan medicine integrating natural sciences, social sciences, and humanities [9, 10]. It summarized the theories, diagnosis, and treatment experience of traditional Tibetan medicine [11–13]. Classic Canon of Mongolian Materia Medica makes a comprehensive summary and some revisions of Mongolian medicine herbal books of the past dynasties [14]. This book records 879 kinds of Mongolian medicinal materials, with 570 medicine illustrations [15]. With the development of Mongolian medicine, the experts of Mongolian medicine actively absorbed the knowledge of plant taxonomy, pharmacognosy, natural pharmaceutical chemistry, pharmacology, and other aspects and compiled a series of teaching materials and authoritative books with the support and organization of the government, such as “Mongolian Pharmacy,” “Encyclopedia of Mongolian Medicine,” “Prescription of Mongolian Medicine,” “Drug standard of the Ministry of health of the people's Republic of China (Mongolian medicine volume),” and “Chinese Materia Medica Mongolian Medicine Volume.” These modern Mongolian medicine books and standards have made important contributions to the modernization of Mongolian medicine and will benefit mankind.

Although both Mongolian medicine and traditional Chinese medicine are traditional medicine, there are many differences in their sources and clinical uses [16, 17]. Mongolian medicine covers a wide range of sources, including plants, animals, minerals, and chemicals [7]. There are 511 kinds of medicinal plants in Mongolian medicine, and 23 species-specific for Mongolian medicine are basal plants, such as *Flos Scabiosae, Lomatogonium rotatum, Dracocephalum moldevica*, etc. [18]. Some medicinal plants are unique to Mongolian medicine and are not available in traditional Chinese medicine [16], such as *Punica granatum* L*., Har Gabur*, *Gardenia jasminoides*, *Syringa pinnatifolia*, *Scabiosa comosa* Fisch, *wannianhui (made from calcareous lumps of ancient buildings)*, *Flos of Aconitum kusnezoffii*, *rhaponticum uniflorum*, *Pearl bar*, *Oxytropis Myriophylla*, etc. Modern pharmacological studies show that Mongolian medicine has good antitumor effects. In the process of chemotherapy, Mongolian medicine can protect normal cells from chemotherapeutic/radiotherapeutic injuries, consolidate or enhance the effect of chemotherapy/radiotherapy, and prevent cancer metastasis and recurrence. This review introduces the ideas of Mongolian medicine in the treatment of liver cancer and the research progress of common clinical drugs (Tables 1 and 2.) and their possible mechanisms.

## 2. Treatment Concept

**Figure 1:**
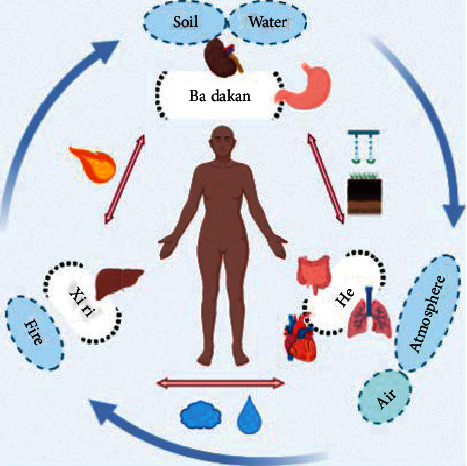
The concept of Mongolian medicine in treating diseases.

Every Chinese Minority established their medical system with their own national characteristics based on the living environment, natural resources, national culture, religious beliefs, and so on, which played key roles in preventing disease and maintaining health [28]. Tibetan medicine, Mongolian medicine, Uygur medicine, Zhuang medicine, Hui medicine, Dai medicine, and Miao medicine are important components of traditional Chinese medicine [29]. The holistic view of the basic theory in Mongolian medicine includes two aspects: the unity of man and nature and the unity of the human body itself. The unity of human beings is the most critical factor in maintaining healthy activities in life [30, 31]. Traditional Mongolian medicine not only accepts Chinese ancient native materialism and dialectics thoughts, Wu yuan, Yin and Yang theory, and absorbs the basic theory of traditional Chinese medicine, but also blends the theory of Tibetan medicine and Indian medicine [32]. In the process of development, it has gradually formed a unique Mongolian medicine theory system, based on the philosophy of Yin and Yang, Wu yuan, Han-re theory; meanwhile, San-gen, Qi-su, San-hui, Zang-fu theory, and Liu-yin theory are considered as the main contents. In the theory of Mongolian medicine, the human body can maintain normal physiological activities mainly because the body has three kinds of energy resources and basic substances, namely, He (equivalent to air), Badakan (equivalent to soil and water), and Xiri (equivalent to fire), which are thought to be the origin and foundation of human life and are also called San-gen. The San-gen theory holds that the life phenomenon of the human body is a comprehensive and complex activity process, in which the organic coordination of San-gen makes the whole life in an orderly metabolic state. The organic connection between the internal organs and body surface tissues is the result of the effects of the San-gen. Therefore, San-gen is the material basis on which life depends (Figure 1).

The concept of Mongolian medicine has gained international attention and has been gradually accepted by people in other parts of the world [33]. The Four Medical Tantras [34] details the etiology, symptoms, diagnosis, treatment, and prevention of the tumor. It regards the human body as an organic whole and considers the tumor as a systemic disease whose occurrence, development, recurrence, and metastasis are the local reflection of systemic diseases. According to different patients, different etiology, different time, the syndrome differentiation for treatment has been done [8].

Mongolian physicians think that the incidence of liver cancer is due to long-term emotional depression, mental traumas, improper diet, long-term addiction to tobacco and alcohol, and traumatic injury. The essence of the human body cannot be normally operated and then accumulates in the liver. As a result, “San-gen” is disordered in vivo and the transportation of blood in the liver is abnormal and deposits [35]. Body fluid, blood stasis, and hot evil then coagulate, throw the organism out of balance, and generate heat which can consistently fumigate the condensation. Ultimately, “Pi kuai” is gradually formed in the liver and the greater the hot evil, the bigger and harder the block. “Pi kuai” is what we call tumors [36].

## 3. Prescription

### 3.1. Qinggan Jiuwei Powder

Qinggan Jiuwei Powder (also named Geiwang-9) is a traditional Mongolian medicine prescription for liver diseases. This prescription was first published in the Four Medical Tantras and then spread to Mongolia and has been used till now. It is recorded in “Encyclopedia of Mongolian Medicine,” “Prescription of Mongolian Medicine.” “Drug Standards of the Ministry of Health of the People's Republic of China (Mongolian Medicines Volume)” [37] and other classic works. This prescription is composed of *Calculus bovis*, *Dianthus superbus*, *trogopterus dung, Scabiosa comosa*, *costus root*, *herpetospermum seed*, *Angelica sinensis*, *Aristolochia manshuriensis*, and *Crocus sativus* [38]. It has the efficacy of cooling blood and removing heat from the liver. Currently, it has been widely used in the treatment of viral hepatitis, cirrhosis, fatty liver, chronic cholecystitis, gastroduodenal ulcer, and other diseases. The experimental research mainly focuses on quality control, technological research, and pharmacological effect. Early diagnosis and treatment of liver fibrosis can effectively improve the life quality of patients and prevent them from developing cirrhosis and even liver cancer [39]. Hepatic fibrosis is caused by excessive deposition of extracellular matrix (ECM) in the liver, which eventually leads to hepatic fibrosis. In normal liver, ECM synthesis and degradation remain dynamically balanced as a result of precise regulation of matrix metalloproteinases (MMPs) and their specific inhibitor, TIMPs [40]. Previous studies [19] have found that Qinggan Jiuwei powder has a certain effect on serum TIMP-1 level and can effectively reduce the imaging index LSM and values measured by abdominal color Doppler ultrasound in patients with alcoholic liver fibrosis [20]. Meanwhile, Qinggan Jiuwei powder [21] can alleviate the symptoms of CCl_4_-induced liver fibrosis in rats, which may be due to its ability to downregulate the serum transaminase and coordinate the MMPs/TIMPs system.

### 3.2. Honghua Qinggan 13 Flavors

Honghua Qinggan 13 flavors (HHQG) is from the Four Medical Tantras and is called “gurigumu-13,” which is included in the “Pharmaceutical Standards of the Ministry of Health of the People's Republic of China” (Mongolian Medicines Volume) [41]. It is composed of *saffron*, *clove*, *lotus seeds*, *Radix Ophiopogonis*, *Radix aucklandiae*, *Melia toosendan*, *gardenia*, *lignum pterocarri*, *musk*, *pulvis cornus bubali concentratus*, *calculus bovis,* and *vermilion* and has the efficacy of clearing heat, detoxifying and cooling blood. At present, there are many studies on the liver-protecting pharmacological effects of single drugs in the prescription but few studies on the pharmacological effects and mechanism of the prescription. HHQG can be used to treat liver diseases [41, 42], including liver failure, drug-induced hepatitis, alcoholic liver, fatty liver, etc. [43]. HHQG exhibits a certain therapeutic effect on liver injury and fibrosis of rats caused by CCl_4_, and the mechanism may be related to the antioxidant effect on regulating the activity of MMP-1 and TIMP-1 [22]. Hepatic fibrosis is the prophase lesion of cirrhosis and the key link in the progression of hepatocellular carcinoma. The anti-inflammation, free radical scavenging, antioxidant, activated HSC apoptosis, immune regulation, antiendotoxin, and other effects of the single drug were analyzed. The main mechanism of its antihepatic fibrosis may be through TGF- 1/Smad and NF-*κ*B signal transduction pathways [23]. Ruimin Li [24] discussed the relationship between hepatitis B virus tumor markers and liver cancer. HHQG can significantly decrease four tumor markers, including fetoprotein (AFP), A-L-fucoidase (AFU), R-glutamate transdermal enzyme (R-GT), and carcinoembryonic antigen (CEA) and reduce HBV-DNA, which indicates a lower cancer rate.

### 3.3. Changpu Siwei

Changpu Siwei is composed of *Galanga Rhizome*, *Halitum Purpureum*, *Vladimiriae Radix,* and *Acorus gramineus*. This prescription has the efficacy of suppressing “Ba dagan and He” and relieving asthma and pain, therefore, being used for oppression in the chest, dyspnea, indigestion, asthma, and pain in clinics [44]. In Mongolia, Changpu Siwei is named Shu da ge-4. It is included in the “Pharmaceutical Standards of the Ministry of Health of China (Mongolian Medicine)” [37]. The Ethanol and petroleum ether extracts of *Changpusiwei* exhibit prominent growth inhibitory effects on SMMC-7721 cells at 12.5∼200 g/mL [25].

### 3.4. Empirical Prescriptions

There are many empirical prescriptions with obvious clinical efficacy [45], such as E ligeng-II, Safflower qinggan 13 flavors, et al. They are effective to treat liver pain and liver enlargement of liver cancer with the function of activating blood circulation, removing blood stasis, and relieving pain [46]. At present, the pharmacological mechanism of most Mongolian prescriptions used in clinics has not been studied. They are used according to pharmacopoeia and physicians' experience. Hepatoprotective Mongolian I and II are two empirical prescriptions commonly used to cure liver cancer.

#### 3.4.1. Hepatoprotective Mongolian I

Hepatoprotective Mongolian I (HM I) is a complex mixture of 18 natural plants in which *Terminalia chebula* is the main ingredient*. Terminalia chebula,* due to its complicated components, has a wide range of pharmacological functions, including prevention and treatment of tumors [47]. Researchers [26] have demonstrated that HM I exhibited significant proliferation inhibition on Huh-7 cells and the mechanism involves cell cycle arrest and cell apoptosis promotion. In addition to the growth inhibition effect, the chemotherapy sensitization effect of HM I was also found. These results have great significance for the popularization and application of HM I against liver cancer in clinics.

#### 3.4.2. Hepatoprotective Mongolian II

Hepatoprotective Mongolian prescription II (MPII) consists of 18 medicinal herbs. It has been reported that MPII [27] significantly inhibited the growth of human liver cancer cells Huh-7 and HepG2. At the molecular level, MPII induced cell apoptosis, arrested G0/G1 cell cycle phase, and promoted expressions of caspase-3, caspase-8, caspase-9, and cytochrome c in Huh-7 and HepG2 cells. *In vivo*, MPII dramatically inhibited human liver cancer growth in a xenograft model in Kunming mice with no apparent cytotoxicity to the hosts. When combined with 5-FU, MPII decreased the toxicity of 5-FU on liver cancer cells. These results have suggested that MPII might have the potential to be a powerful therapy in liver cancer.

## 4. Monomer Compounds and Extracts

Commonly used medicines for removing stasis and stagnation in Mongolian medicine include *Monetariae concha*, *Concha Mauritiae*, *Clematis aethusifolia Turcz.*, *Clematis intricata Bunge*, *Ranunculus sceleratus L.*, *Parnassia palustris L.*, *GnaphaliumaffineD. Don,* etc. They are used to treat food stagnation, all kinds of “Pi kuai,” carbuncle swelling caused by metabolic disorders, and the accumulation of scum and essence [35]. Here, we summarize the common drugs and monomers for the treatment of liver cancer.

### 4.1. *Terminalia chebula* (*Terminalia chebula Retz*)

**Figure 2:**
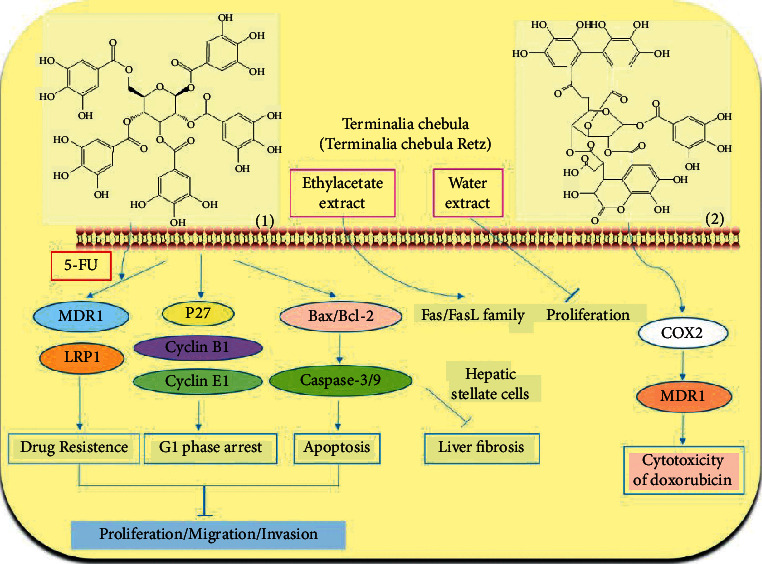
The anticancer mechanism of *Terminalia chebula* in liver cancer.

*Terminalia chebula* is the dried ripe fruit of *Terminalia Chebula Retz.* or *Terminalia Chebula Retz. var. tomentella Kurt* which belongs to the family of *Combretaceae R. Br*. It is not only used in Mongolian medicine, Chinese medicine, and Tibetan medicine in China but also used in other countries such as India and Iran. It is included in the “pharmacopoeia of the People's Republic of China,” which has the efficacy of restraining “Xiri,” astringent trauma, promoting tissue regeneration, and assisting digestion, and detoxication [35]. Mongolian doctors believe that *Terminalia chebula* can cure all kinds of toxicities [48]. Modern pharmacological research has shown that *Terminalia chebula* can be used for asthma, inflammation, neurological disorders, and wound infection and be frequently used as a part of many preparations to treat a variety of diseases; therefore, it is known as “the king of medicine” [49]. Pentagalloyl glucose (PGG, 1) is a natural polyphenol from *Terminalia chebula*. It has been reported that PGG has anticancer activity in ovarian cancer and nasopharyngeal carcinoma [50]. Researchers [51] have suggested that PGG can inhibit the proliferation, migration, and invasion of HepG2 cells and induce cell cycle G1 phase arrest and cell apoptosis. The combination of PGG and 5-FU shows a synergistic effect on the reversal of the aggressive phenotypes of HepG2 cells. PGG has the potential to be used to treat liver cancer in clinics. Chebulagic acid (2) is a benzopyran tannin obtained from *Terminalia chebula*. The combination of Chebulagic acid and doxorubicin shows strong synergism in inhibiting liver cancer cell growth. Furthermore, Chebulagic acid can enhance the sensitivity of HepG2 cells to doxorubicin, thus showing anticancer effects against liver cancer [52]. Chen et al. [53] have found that ethyl acetate extract of *Terminalia chebula* can mediate the gene expression of Fas/FasL family through an exogenous pathway, thus inducing the apoptosis of immortalized rat hepatic stellate cells. Reversing liver fibrosis can effectively prevent the development of liver cancer [54]. The ethyl acetate extract of *Terminalia chebula* can effectively reverse the development of liver fibrosis and, to some extent, prevent liver cancer. The water extract of *Terminalia chebula* can obviously inhibit the proliferation activity of liver cancer cells [55] (Figure 2).

### 4.2. Safflower (*Carthamus tinctorius* L.)

Safflower is the dry flower of *Compositae Carthamus tinctorius L*. The Mongolian medicine canonical “Classic Canon of Mongolian Materia Medica” [56] has recorded that safflower can remove liver heat, regulate menstruation, detumescence, and stop bleeding. It has long been used in Mongolian medicine and traditional Chinese medicine [57, 58]. This herbal medicine is clinically compatible with other drugs for the treatment of hepatomegaly, liver damage, and irregular menstruation. Safflower yellow B (SYB, 3) is one of the main bioactive constituents of safflower. Sharula et al. [59] have proposed SYB to be a promising therapeutic compound for liver cancer as they found that SYB inhibited cell proliferation and promoted cell apoptosis mainly through miR-34a/P53/caspase-9 axis in HepG2 cells, demonstrating the clinical application value of SYB in liver cancer treatment.

**Figure 3:**
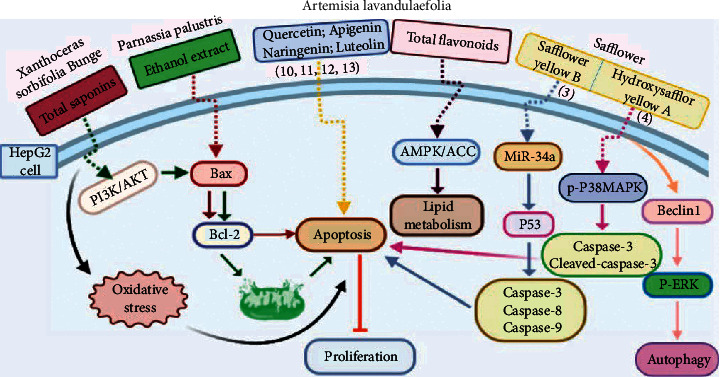
The anticancer mechanism of monomer compounds and extracts in liver cancer.

Hydroxysafflor yellow A (HSYA, 4), a water-soluble chalcone from safflower, is frequently studied previously for its neuroprotective effect in cerebrovascular and neurodegenerative diseases. Recently, the positive action of HSYA in the prevention of liver damage caused by chemicals or alcohol and the anticancer effect of HSYA in various types of cancers are also reported. HSYA inhibited proliferation, migration, and induced apoptosis through suppressing p38MAPK signaling in HepG2 cells [60]. Another research has shown that HSYA induced autophagy by promoting the expression of Beclin 1 and inhibited the phosphorylation of ERK in liver cancer cells [58]. These findings provide experimental evidence that HSYA might be a promising anticancer agent for HCC (Figure 3).

### 4.3. *Xanthoceras sorbifolia* Bunge

*Xanthoceras sorbifolia* Bunge, which belongs to Sapindaceae, has been used as TCM for curing arterial sclerosis, hyperglycemia, hyperpiesia, chronic hepatitis, rheumatism, and enuresis of children [61, 62]. The chemical constituents and pharmacological activities of its branches, leaves, flowers, stalks, kernels, shells, and wood have been studied by scholars at home and abroad. The wood, branches, and leaves have been used in Mongolian medicine and are thought to have significant effects on drying “Xiri,” clearing heat, and relieving swelling and pain. Lili Yu [61] isolated some triterpenoid saponins from the seed oil leavings of *X. sorbifolia* Bunge and found cytotoxicity of these compounds on several human cancer cell lines. Compounds 5 (IC_50_ = 2.45 ± 0.58 *μ*M) and 7 (IC_50_ = 4.03 ± 0.75 *μ*M) show significant activity against HepG_2_ cell line, while compounds 6 (IC_50_ = 22.20 ± 1.92 *μ*M), 8 (IC_50_ = 60.83 ± 0.94 *μ*M) and 9 (IC_50_ = 33.11 ± 2.21 *μ*M) exhibit moderate activity against HepG2 cell line (Table 2). Extracts from the *X. sorbifolia* Bunge exhibit cytotoxicity toward various human cancer cell lines [63, 64]. Meanwhile, TSXS could lead to apoptosis by stimulating the cells to produce oxidative stress (Figure 3). Total saponins from *X. sorbifolia* Bunge (TSXS) induced apoptosis of HepG2 cells through mitochondria-mediated apoptosis pathway and arrested the cell cycle at the S phase [63]. Polyphenols from the Husks of *X.Sorbifolia* exhibited anticancer and radical-scavenging effects in several cancer cells [64]. The results of these studies provide a theoretical basis for further development of *X. sorbifolia* Bunge.

### 4.4. *Parnassia palustris*

Parnassia palustris is the whole herb of *Parnassia palustris* Linn. It is commonly used for clearing heat, detoxifying, reducing swelling and eliminating abscess during the treatment of various diseases, such as Jaundice hepatitis, laryngitis, mumps, vasculitis, tuberculosis, and cancers [65, 66]. It has been demonstrated that the ethanol extract of *Parnassia palustris* significantly inhibited the proliferation of HepG2 cells and, by detecting apoptosis-related proteins, the underlined mechanism might involve apoptosis promotion [67, 68] (Figure 3).

### 4.5. *Artemisia lavandulaefolia DC*

*Artemisia lavandulaefolia DC*. is used in traditional Mongolian medicine as a perennial herb that is widely distributed in Inner Mongolia of China. It is a member of Artemisia compositae, with expelling cold, clearing damp, warming, and activating meridian, halting bleeding, antibacteria, antiallergy, anticancer effects, and it is commonly used in formulae, such as TGLG-1 [69, 70]. Modern pharmacology research has shown that extracts and compounds from *A. lavandulaefolia* exhibited potential anticancer activities. Quercetin (10) and apigenin (11) which are isolated from *A. lavandulaefolia* [71] displayed apoptosis promotion effect in HepG2 cells. Meanwhile, four kinds of flavonoid glycosides, including luteolin (12), naringenin (13), quercetin, and apigenin, from *A. lavandulaefolia* could inhibit the proliferation of HepG2 cells [72]. The total flavonoids of *A. lavandulaefolia* activated the AMPK-ACC pathway in HepG2 cells, therefore participating in the lipid metabolism of HepG2 cells [73] (Figure 3).

### 4.6. Flowers of *Hosta plantaginea*

Hosta is a genus of the Liliaceae family. There are four primitive species in China, *H. plantaginea*, *H. ventricosa*, *H. ensata*, and white *H. albofarinosa* [74]. *H. plantaginea* is a landscaping plant and an annual herb widely distributed in temperate and subtropical Asia, including China, Japan, North Korea, and the far east of Russia, mostly cultivated. The dried flowers of *Hosta plantaginea* as Mongolian medicine were documented in “Chinese Materia Medica Mongolian medicine roll.” It possesses diuresis detumescence, heat-clearing, and detoxicating, hemostasis, and anti-inflammatory effects [48]. *H. ventricosa* was also recorded in “Chinese Materia Medica Mongolian medicine roll,” with cooling blood and hemostasis and detoxicating effects. The whole plant of *H. ventricosa* is used as medicine [37]. Studies [75] have indicated that the total saponins of *H. ventricosa* (TSHV) could effectively inhibit the proliferation of HepG2 cells *in vitro* with an IC_50_ value as 17.37 *μ*g/L. Pharmacological and chemical studies on *H. plantaginea* have suggested that the alcohol extract and some compounds showed antibacterial [76], ant-fungal [77], anti-inflammatory [78, 79], analgesic [80], and anticancer effects [78], etc. The cytotoxic effect of four monomer compounds isolated from the flowers of *H. plantaginea* has been studied [81]. Among these compounds, compound Gitogenin (14) selectively inhibited the proliferation of cancer cells, including K562, YAC-1 and SMMC-7721 cell lines, and the IC_50_ of compound (14) in SMMC-7721 is 2.84 *μ*g/ml. The steroidal saponins of *H. plantaginea* showed cytotoxicity on various cancer cell lines and these anticancer effects are cell type dependent [82]. Compounds (15), (16), (17), (18), and (19) displayed strong inhibitory effects on human liver cancer cells (HepG2) with IC_50_ values ranging from 0.16 mol/L to 1.16 mol/L, which are equivalent to or stronger than the effect of cisplatin, a positive drug in the experiment. Wei et al. [83] isolated and identified some flavonoids from *H. plantaginea* (Lam.) Aschers. They examined the hepatoprotective activity of these flavonoids on CCl_4_-induced injury of human L-O2 cells and found that compounds (20), (21), (22), and (23) exhibited moderate hepatoprotective activities.

### 4.7. *Artemisia frigida Willd*

*Artemisia frigida* belongs to *Artemisia* (Compositae). It has been used for hundreds of years as a Mongolian traditional herbal medicine which is widely distributed in the Inner Mongolia Autonomous Region of China [84]. It is also named Xiaobaihao or Hanhao in Chinese and “Agi” in Mongolian. After flowering, the aboveground part of *A. frigida* is harvested, dried in the shade, and ground into powder. It is used for the treatment of various bleeding, kidney fever, irregular menstruation, sores, and Carbuncle pains [85, 86]. Researchers [87] have found five sesquiterpenoids from *A. frigida* and these compounds exhibited obvious inhibitory effects on human cancer cells. One of the compounds, dehydrocostuslactone (24) exhibited significant inhibition in HLE cells with the IC_50_ of 22.50 *μ*M/L. Dihydrodehydrocostuslactone (25) possessed moderate inhibition in human cancer cells only in high concentration treatment. Unsaturated lactone is the essential functional group of sesquiterpene lactones which inhibited the proliferation of cancer cells.

### 4.8. Sea Buckthorn (*Hippophae rhamnoides L.*)

**Figure 4:**
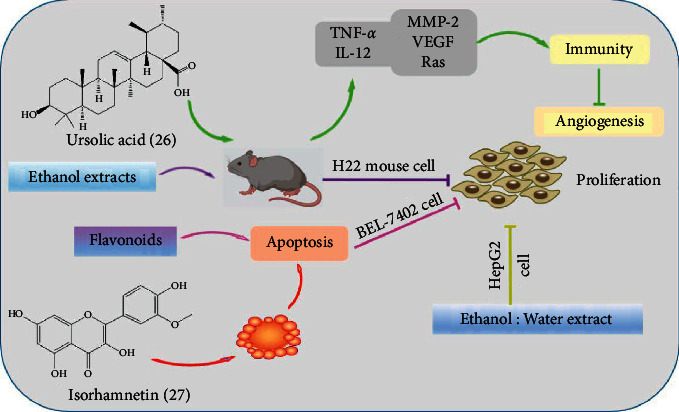
The anticancer mechanism of Sea buckthorn in liver cancer.

Sea buckthorn is the fruit of *Hippophae rhamnoides* L. which is a wild berry plant characterized by multiple economic advantages and versatile properties. *H. rhamnoides* L. is a kind of “medicine and food homologous” plant with high utilization value. Sea buckthorn is commonly used in Mongolian medicine to cure cough, sputum, pulmonary tuberculosis, pulmonary abscess, and lung cancer according to its effects of relieving cough, eliminating phlegm, suppressing “Badakan,” and promoting digestion. Sea buckthorn consists of more than 100 kinds of bioactive compounds, including essential amino acids, vitamins, trace elements, tocopherols, carotenoids, polyphenols, flavones, and other active substances [48, 88]. Ursolic acid (26), extracted from *H. rhamnoides* L., has been reported to increase IL-12 and TNF-*α* [89], activate cell immunity, and further inhibit angiogenesis, finally showing anticancer effect against liver cancer in vivo. Grey et al. [90] have performed a sequential extraction and found that ethanol: water extract of sea buckthorn exerted the strongest proliferation inhibitory effect in HepG2 cells. The 80% ethanol reflux extracts of sea buckthorn fruit, stem, and leaf showed different degrees of anticancer effect on mouse hepatocellular carcinoma H22 tumor strains [91]. Flavonoids from oil-removed seeds of *H. rhamnoides* (FSH) could inhibit cell proliferation and induce cell apoptosis in a dose-dependent manner (200∼1200 *μ*g/mL) in BEL-7402 cells [92]. Isorhamnetin (27), a flavonol aglycone, was isolated from *H. rhamnoides* L. and showed cytotoxicity on BEL-7402 cells dose- and time-dependently, with IC_50_ of 74.4 ± 1.13 *μ*g/ml [93]. The mechanism of this cytotoxicity in response to isorhamnetin (50 *μ*g/ml, 48 h) might involve its apoptosis promotion effect (Figure 4).

## 5. Discussion

Currently, the progress of liver cancer clinical treatment has been few and far between. Due to the rapid development of the disease, most of the patients are in the middle and late stage or even have metastases at the time of clinical diagnosis and could not be treated with radical resection [94]. Therefore, it is urgent to find more promising treatment strategies.

Mongolian medicine is not only an important part of Chinese traditional medicine but also a treasure of Chinese traditional culture and national intangible cultural heritage. It has important medicinal and academic value. The main drugs (prescriptions) used in the treatment of tumors in Mongolian medicine contain ruyijiedu pill, jisiwunisi-25 pill, hualiu pill, Zhuangxi Power, gamujur, habuder-9 powder, susi-12 powder, etc. Naren mandu la -11 [95, 96] and Aili gen-II are clinical prescriptions commonly used to treat liver cancer and have been proved to be effective in liver cancer therapy. Notably, the research on Mongolian medicine mostly stays at the original treatment level, while the research on the underling mechanism is relatively less. Most of the studies focus on the anticancer activities of monomer compounds obtained from Traditional Mongolian Medicine. However, studies on the activity and mechanism of formulae are few. Furthermore, most experiments evaluating anticancer mechanism of Traditional Mongolian Medicine are just conducted on cancer cell models in vitro. The proofs in vivo are limited [6]. At present, the bottleneck in the development of Mongolian medicine lies in the fact that Mongolian medicine is not well known by the vast majority of people in China so it just plays roles in a relatively small scope. Therefore, further in-depth research of the anticancer mechanism of Mongolian medicine in liver cancer and close combination of the basic research with clinical application is urgently required.

## 6. Conclusions

Mongolian medicine has been used for a long history to prevent or treat various diseases, including liver cancer. It shows mild and lasting pharmacological effects with multiple advantages: amelioration of sensory symptoms, improvement of the quality of life, prolongation of overall survival and, to some extent, low side effects. This review provides a comprehensive summary of Traditional Mongolian Medicine, with the purpose of finding promising drugs to treat liver cancer and expanding the clinical application of Traditional Mongolian Medicine in liver cancer therapy.

## Figures and Tables

**Table 1:** Prescriptions for the treatment of liver cancer in Mongolian medicine.

Prescription	Components	Treatment concept	Cell model	Animal model	Pharmacologic action	Clinical application	Reference
Qinggan jiuwei powder	Calculus bovis, *Dianthus superbus*, *Trogopterus* dung, *Scabiosa comosa*, *Costus* root, *Herpetospermum* seed, *Angelica sinensis, Aristolochia manshuriensis, Crocus sativus*	Removing heat from the liver; cooling blood	—	CCl_4_-induced liver fibrosis in rats	Suppress or alleviate liver fibrosis	Viral hepatitis, cirrhosis, fatty liver, chronic cholecystitis, gastroduodenal ulcer, etc.	[19–21]
Honghua qinggan 13 flavors	Saffron, clove, lotus seeds, radix ophiopogonis, radix aucklandiae*, Melia toosendan, Gardenia, Lignum pterocarri,* musk, pulvis cornus bubali concentratus, calculus bovis, and vermilion	Clearing heat; detoxifying; cooling blood	—	CCl4-induced liver fibrosis in rats	Liver injury and fibrosis	Liver failure, drug-induced hepatitis, alcoholic liver, fatty liver, etc.	[22–24]
Changpu siwei	Galanga rhizome, *Halitum purpureum*, vladimiriae radix, and *Acorus gramineus*	Suppressing “ba dagan and he”; relieving asthma and pain	SMMC-7721 cell	—	Inhibition the proliferation of liver cancer cells	Oppression in chest and dyspnea, indigestion, relieving asthma and pain	[25]
Hepatoprotective Mongolian I	*Terminalia chebula* is the main component	Activating blood circulation, removing blood stasis, and relieving pain	Huh-7 cell	—	Inhibition the proliferation of liver cancer cells, inducing apoptosis	Prevention and treatment of tumors	[26]
Hepatoprotective Mongolian II	*Terminalia chebula* is the main component	Activating blood circulation, removing blood stasis, and relieving pain	Huh-7, HepG2 cell	—	Inducing apoptosis and cell cycle arrest	Prevention and treatment of tumors	[27]

**Table 2:** Monomer compounds against liver cancer in the Mongolian medicine.

	Chemical name	Botanical name	Compound structure	Structure type
1	Pentagalloyl glucose	*Terminalia chebula* retz	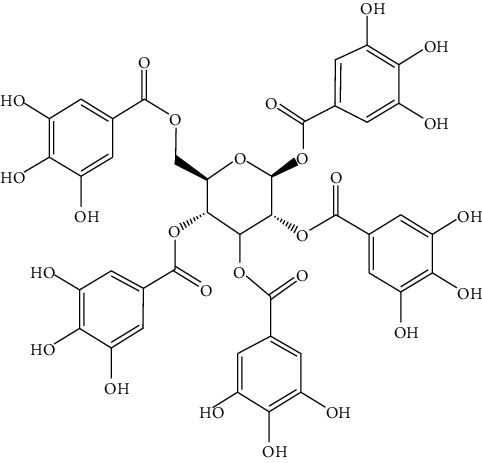	Polyphenols
2	Chebulagic acid	*Terminalia chebula* retz	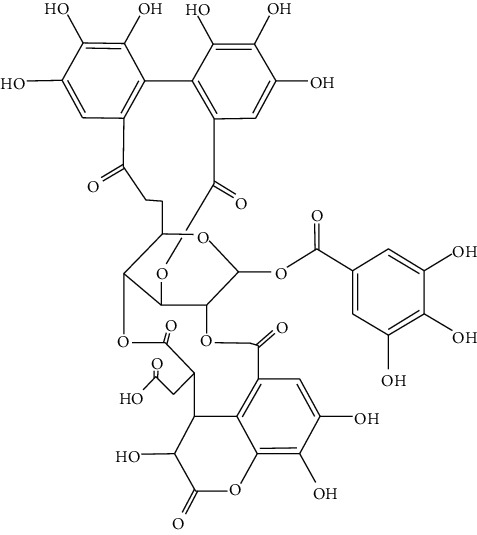	Phenols
3	Safflower yellow B	*Carthamus tinctorius* L.	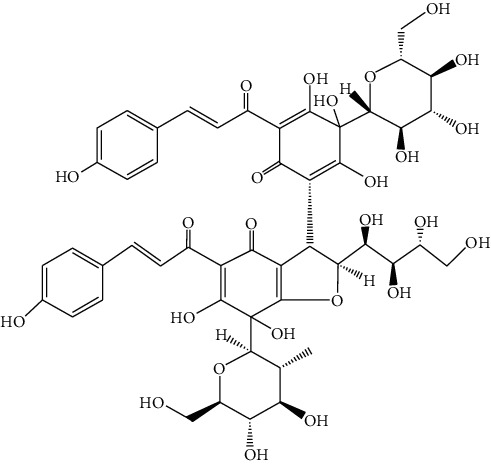	Flavonoid
4	Hydroxysafflor yellow A	*Carthamus tinctorius* L.	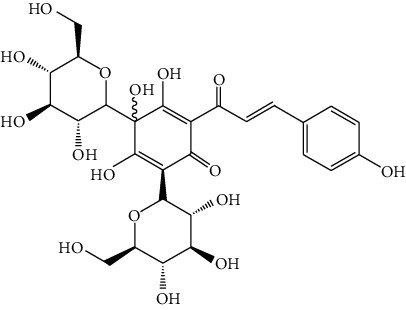	Flavonoid
5, 6, 7, 8, 9	—	*Xanthoceras sorbifolia* Bunge	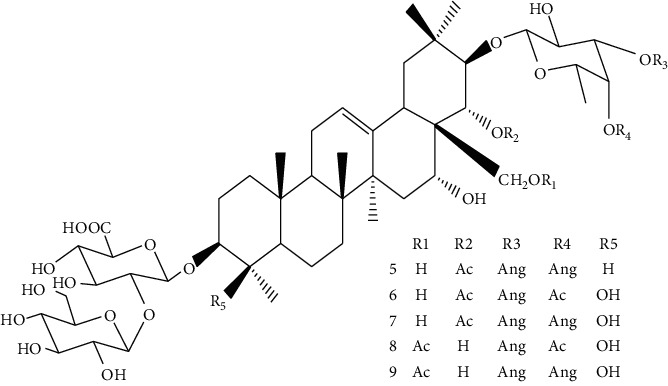	Triterpenoid saponins
10	Quercetin	*Artemisia lavandulaefolia* DC.	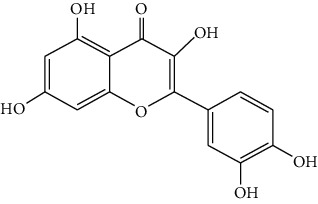	Flavonoid
11	Apigenin	*Artemisia lavandulaefolia* DC.	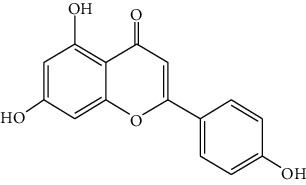	Flavonoid
12	Luteolin	*Artemisia lavandulaefolia* DC.	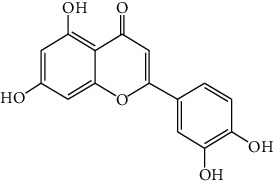	Flavonoid
13	Naringenin	Artemisia lavandulaefolia DC.	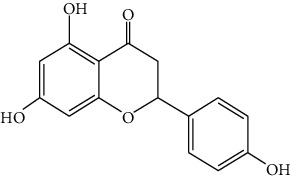	Flavonoid
14	Gitogenin	*Hosta plantaginea*	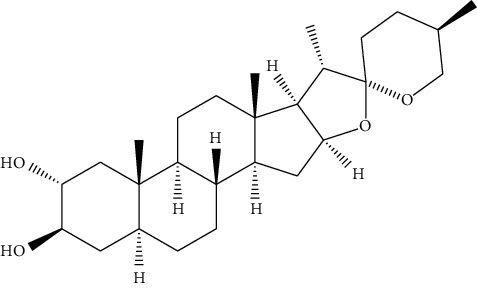	Steroidal saponin
15	Gitogenin3-O-*β*-dglucopyranosyl (1⟶2)-*β*-D-glucopyranosyl (1⟶4)-*β*-D-galactopy ranoside	*Hosta plantaginea*	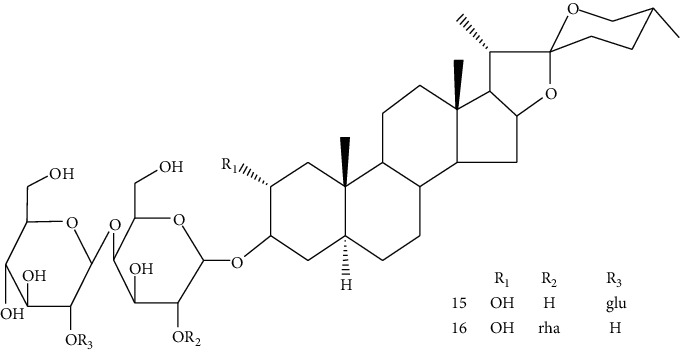	Steroidal saponin
16	Gitogenin3-O-*β*-D-glucopyranosyl (1⟶4)-O-[*α*-L-rhamnopyranosyl (1⟶2)]-*β*-D-galactopyranoside	*Hosta plantaginea*		Steroidal saponin
17	Gitogenin3-O- {*β*-D-glucopyranosyl (1⟶2)-O- [*β*-D-xylopyranosyl (1⟶3)]-O-*β*-D-glucoyranosyl(1⟶4)-*β*-D-galactopyranoside}	*Hosta plantaginea*		Steroidal saponin
18	Gitogenin3-O- {*β*-D-glucopyranosyl (1⟶2)-O- [*α*-L-rhamnopyranosyl (1⟶4)-*β*-D-xylopyranosyl(1⟶3)]-O-*β*-D-glucopyranosyl (1⟶4)-*β*-D-galactop ranoside}	*Hosta plantaginea*		Steroidal saponin
19	Gitogenin 3-O-{*β*-D-xylopyranosy l(1⟶4)-*β*-D-glucopyranosyl (1⟶2)-[*β*-D-xylopy ranosyl (1⟶3) ]-O-*β*-D-glucopyranosyl(1⟶4)-*β*-D-galactopyranoside}	*Hosta plantaginea*	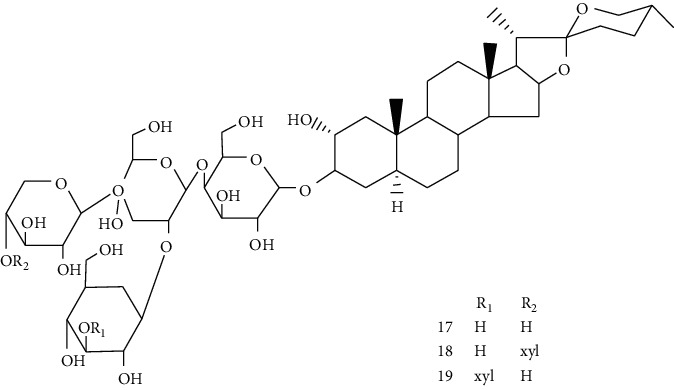	Steroidal saponin
20	**—**	*Hosta plantaginea*	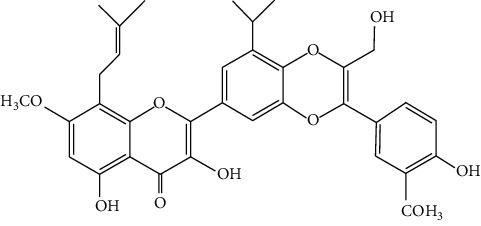 3	Flavonoid
21	**—**	*Hosta plantaginea*	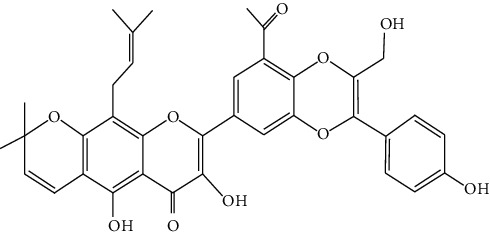	Flavonoid
22	**—**	*Hosta plantaginea*	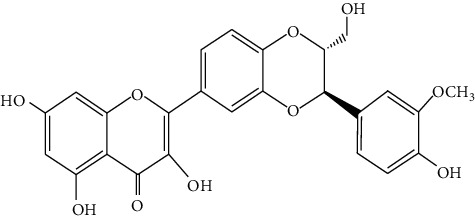	Flavonoid
23	**—**	*Hosta plantaginea*	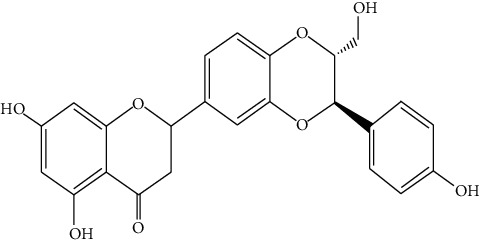	Flavonoid
24	Dehydrocostuslactone	*Artemisia frigida willd*	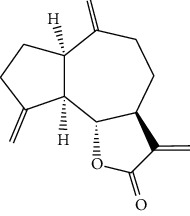	Sesquiterpenoid
25	Dihydrodehydrocostuslactone	*Artemisia frigida willd*	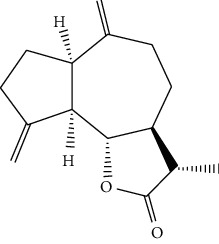	Sesquiterpenoid
26	Ursolic acid	*Hippophae rhamnoides* L.	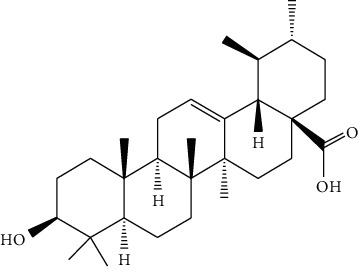	Triterpenoid
27	Isorhamnetin	*Hippophae rhamnoides* L.	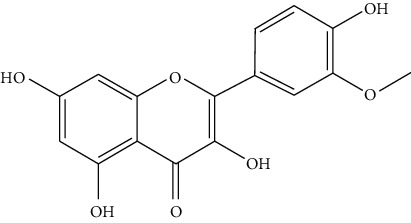	Flavonol aglycone

## Data Availability

All data used to support the findings of this study are included within the article.
